# Supplementary material for: Whole blood DNA methylation analysis reveals respiratory environmental traits involved in COVID-19 severity following SARS-CoV-2 infection
Source: Nat Commun. 2022 Aug 6;13:4597. doi: 10.1038/s41467-022-32357-2 (PMC9357033; doi:10.1038/s41467-022-32357-2)
Supplement: Supplementary file 1 — Supplementary Information [file 41467_2022_32357_MOESM1_ESM.pdf]

Whole Blood DNA Methylation Analysis Reveals Respiratory Environmental Traits Involved in COVID-19 Severity Following SARS-CoV-2 Infection

Guillermo Barturen*, Elena Carnero-Montoro, Manuel Martínez-Bueno, Silvia Rojo-Rello, Beatriz Sobrino, Óscar Porras-Perales, Clara Alcántara-Domínguez, David Bernardo and Marta E. Alarcón-Riquelme*

(Supplementary Information)

Supplementary Figures

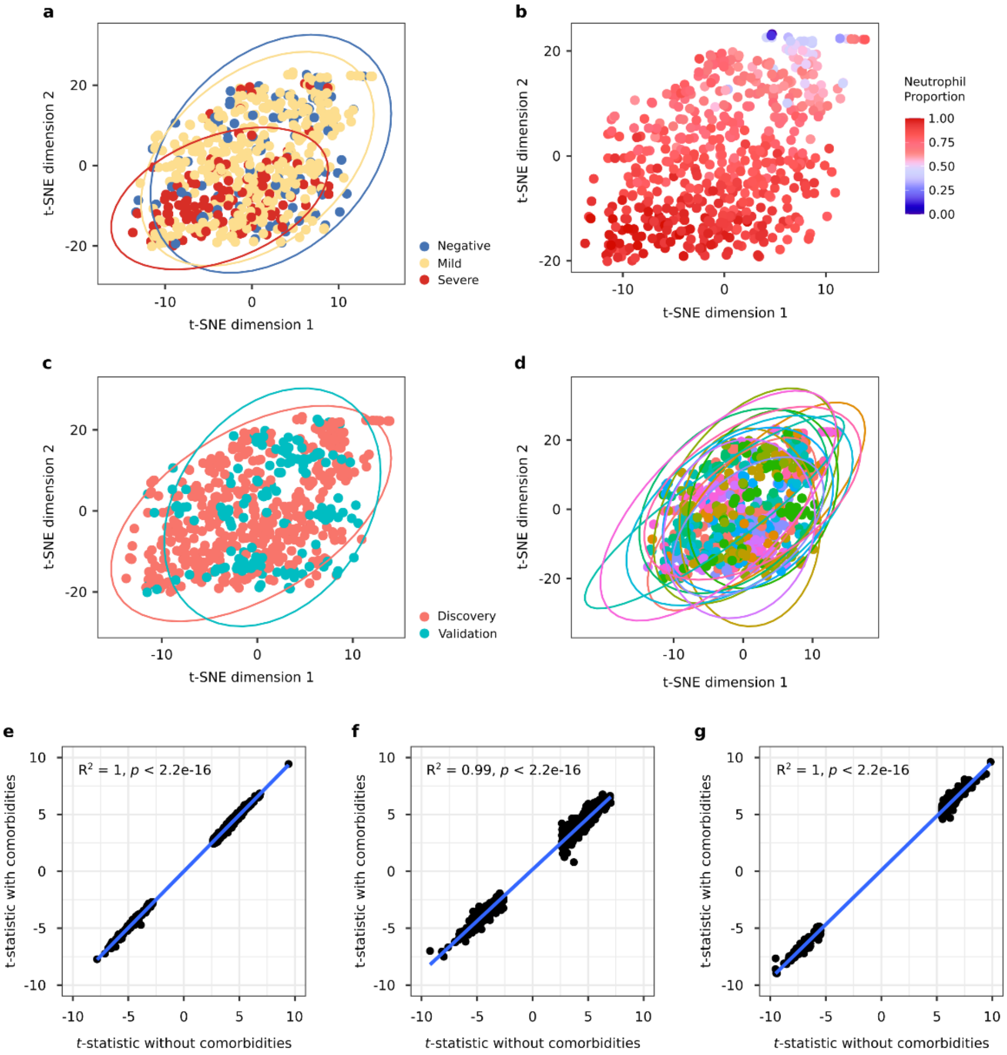

Supplementary Figure 1: Technical batch effect and comorbidities did not bias the methylation profiles. t-SNE analysis of the 10.000 most variable CpGs (based on the DNA methylation absolute deviation mean) is shown colored by different variables: (A) severity groups (Severe, mild and negative individual groups are colored in red, yellow and blue respectively), (B) neutrophil proportion, (C) cohorts (discovery in red and validation in green) and (D) technical batch. Discovery (E), replication (F) and meta-analysis (G) statistics correlated with and without including the comorbidities in the regression models.

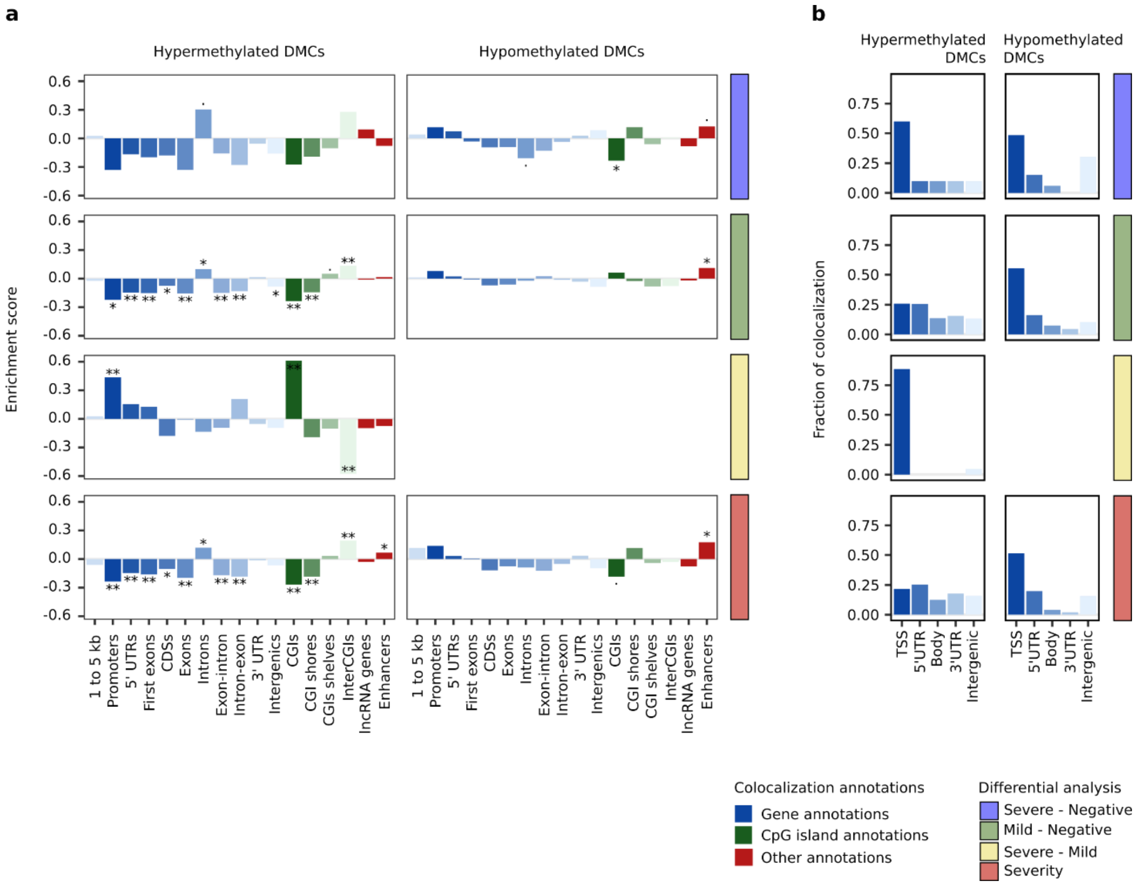

Supplementary Figure 2: Hypomethylated and hypermethylated DMCs are mostly enriched and colocalized with gene regulatory elements, which tend to activate and inactivate in *cis* gene expression levels. (A) Significant DMCs enrichment from each differential analysis across different regulatory elements (annotatr R package). Hypermethylated and hypomethylated DMCs are divided into left and right panels, respectively. Each DMC is allowed to be annotated in more than one of the following features: 1 to 5kb, region between 1-5kb upstream from the TSS; promoters, region at less than 1kb upstream, from the TSS; 5' UTR region; first exon; CDS, protein coding regions; exon; intron; exon-intron boundaries; intron-exon boundaries; 3' UTR region; intergenic, not colocalized with any gene annotation (gene annotations colored in blue); CGI, CpG island; CGI shores, at less than 2kb of a CGI; CGI shelves, at 2-4kb of a CGI; interCGI, not colocalized with any CGI annotation (CGI annotations colored in green); lncRNA genes, GENCODE long non-coding gene annotation and enhancer, colocalized with FANTOM5 enhancer database annotation (other annotations colored in red). Enrichment score is defined as the log2FC between the fraction of colocalized DMCs and the CpGs in the EPIC array. Significance was calculated by means of a two-tailed Fisher exact test ($^{\cdot}$*p*-value < 0.05, **p*-value < 0.01 and ***p*-value < 1e-5). (B) Fraction of colocalized DMCs by differential analysis for ranked gene features obtained from the EPIC array annotation (each DMC is assigned to one feature according to: TSS, transcriptions start site > 5' UTR > 3' UTR > Body, gene body not in the previous features > Intergenic, not assigned to any gene). Severe vs negative (blue), mild vs negative (green), severe vs mild (yellow) and pseudotime longitudinal analysis (red).

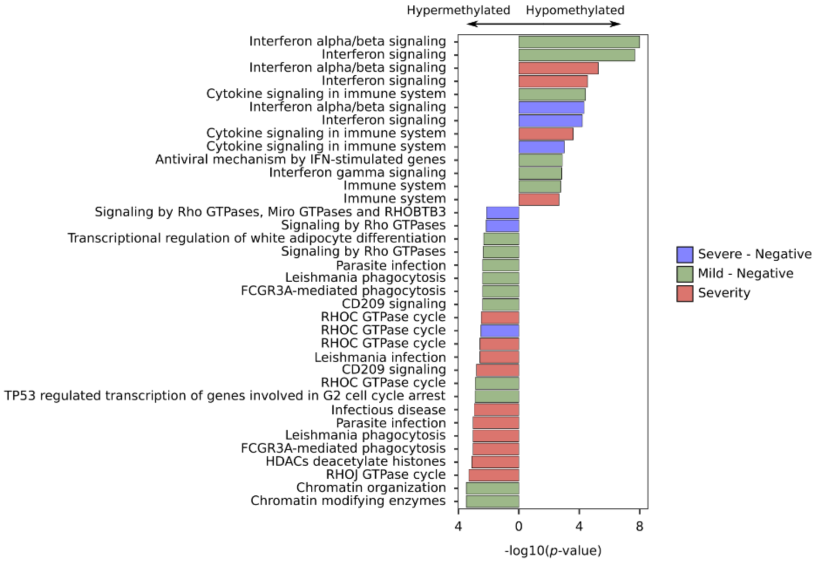

Supplementary Figure 3: CpG probe-oriented enrichment analysis. Top 20 significant reactome database pathways (two-tailed hypergeometric *p*-value < 0.01) obtained by differential analysis are shown. Severe vs negative (blue), mild vs negative (green) and pseudotime longitudinal analysis (red).

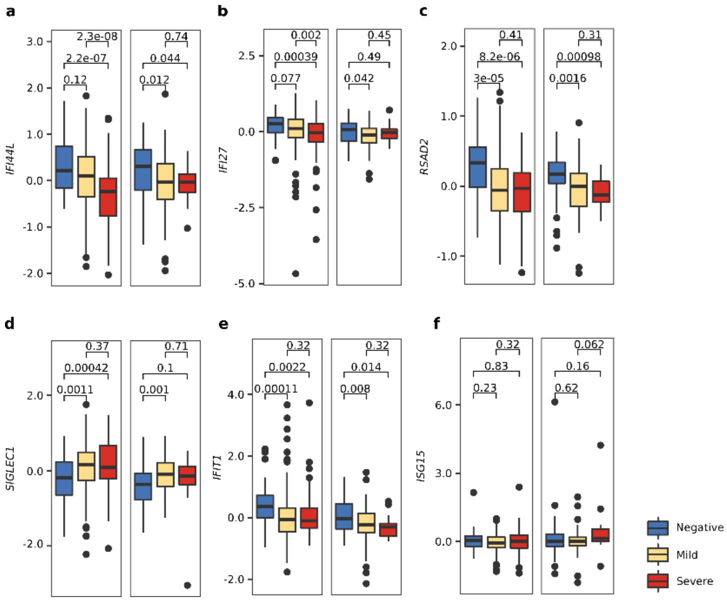

Supplementary Figure 4: Interferon exhaustion in severe COVID-19 patients is not regulated by DNA methylation changes. (A-F) DNA methylation z-scored levels for CpGs colocalized with interferon gene signature promoters are shown by COVID severity group in discovery and replication cohorts. Two-tailed Wilcoxon test *p*-values are depicted by pairs. Blue, 47 and 54 negative SARS-CoV2 lab tested individuals for discovery and replication; yellow, 269 and 91 positive individuals with mild symptoms for discovery and replication and red, 98 and 15 positive individuals with severe symptoms for discovery and validation. The center line denotes the median value, the box contains 25th to 75th percentiles of the dataset and the whiskers extend up to ±1.5*IQR.

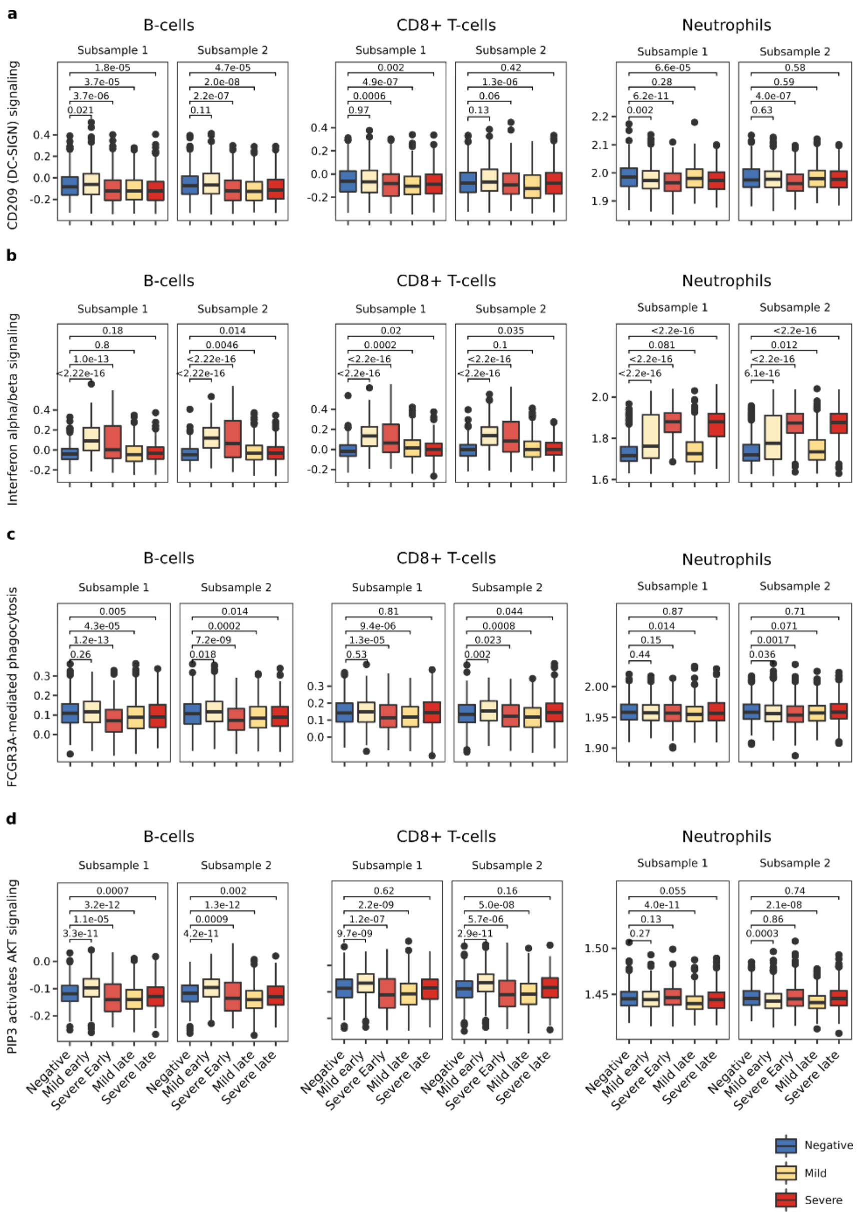

Supplementary Figure 5: Enriched pathway activity in the CpG modules follow DNA methylation changes at early SARS-CoV-2 samplings in the cell-types with significant interactions. Reactome CD209 signaling (A), interferon alpha/beta signaling (B), FCGR3A-mediated phagocytosis (C) and PIP3 activates AKT signaling (D) activities were calculated per individual with ssgsea R package and grouped by COVID-19 severity groups at early and late samplings (>11 days after first symptoms) for B-cells, CD8+ T-cells and Neutrophils. Activities were plotted for two randomly selected subsets of 2500 cells, 500 cells per group. For B-cells and CD8+ T-cells coming from 15 negative controls, 11 mild early, 5 severe early, 30 mild late and 19 severe late patients. For neutrophils coming from 13 negative controls, 2 mild early, 1 severe early, 3 mild late and 4 severe late. Two-tailed Wilcoxon test *p*-values are depicted against healthy controls. The center line denotes the median value, the box contains 25th to 75th percentiles of the dataset and the whiskers extend up to ±1.5*IQR. Severe, mild and negative individual groups are colored in red, yellow and blue respectively.

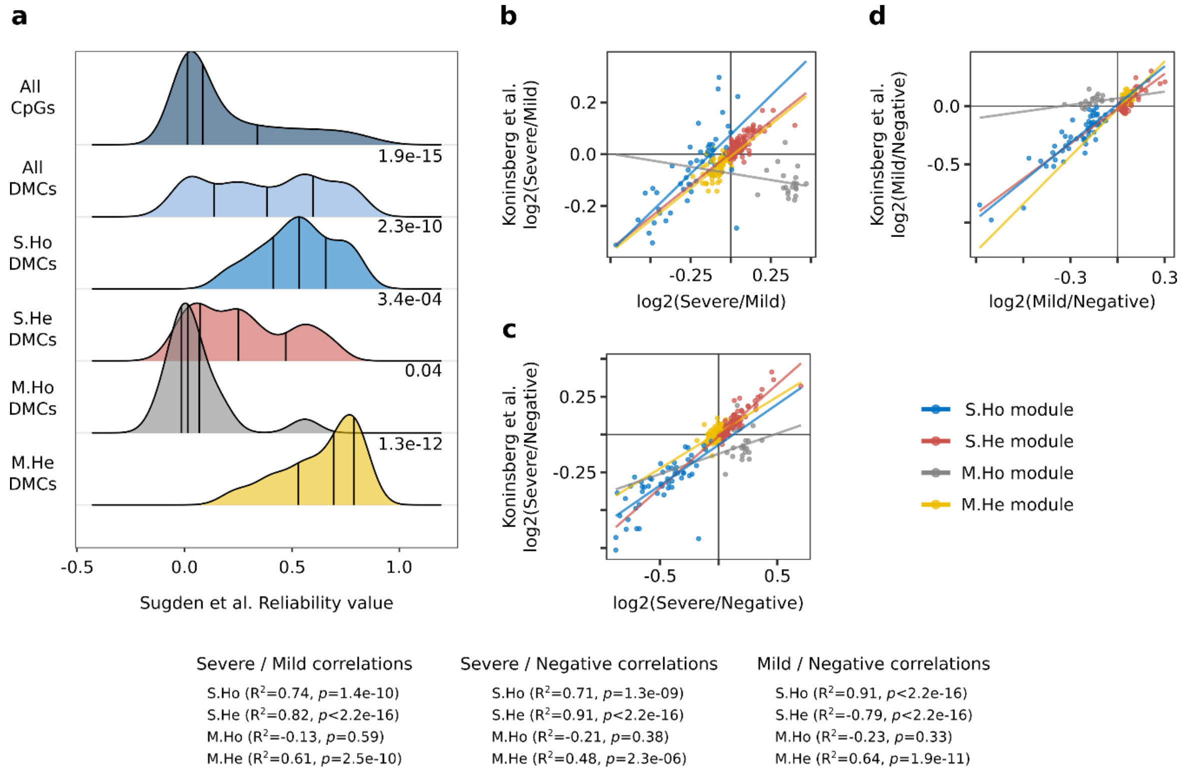

Supplementary Figure 6: CpG module reliability tests. (A) Sugden et al. reliability density metric distribution is plotted for all CpG probes (dark blue), DMC probes (light blue) and probes within CpG modules S.Ho (blue), S.He (red), M.Ho (grey) and M.He (yellow). Vertical lines in the distributions represent percentiles 25, 50 and 75. Each distribution is compared with all CpG probes distribution by means of Kolmogorov-Smirnov test and the *p*-value depicted. Log2 fold-changes of (B) severe versus mild, (C) severe versus negative and (D) mild versus negative methylation values of our cohorts are compared with an external dataset from Koninsberg et al. Individual probe log2 fold-changes are plotted and colored by CpG module membership.

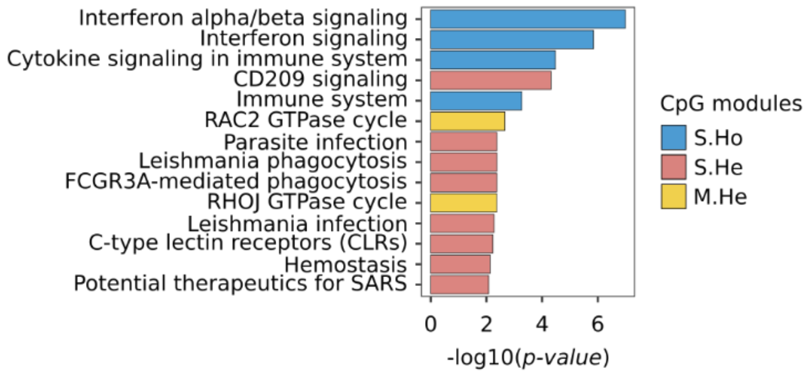

Supplementary Figure 7: CpG probe-oriented enrichment analysis by module. Reactome significant pathways by CpG module (two-tailed hypergeometric *p*-value < 0.01), are shown. S.Ho module is colored in blue, S.He in red and M.He in yellow.

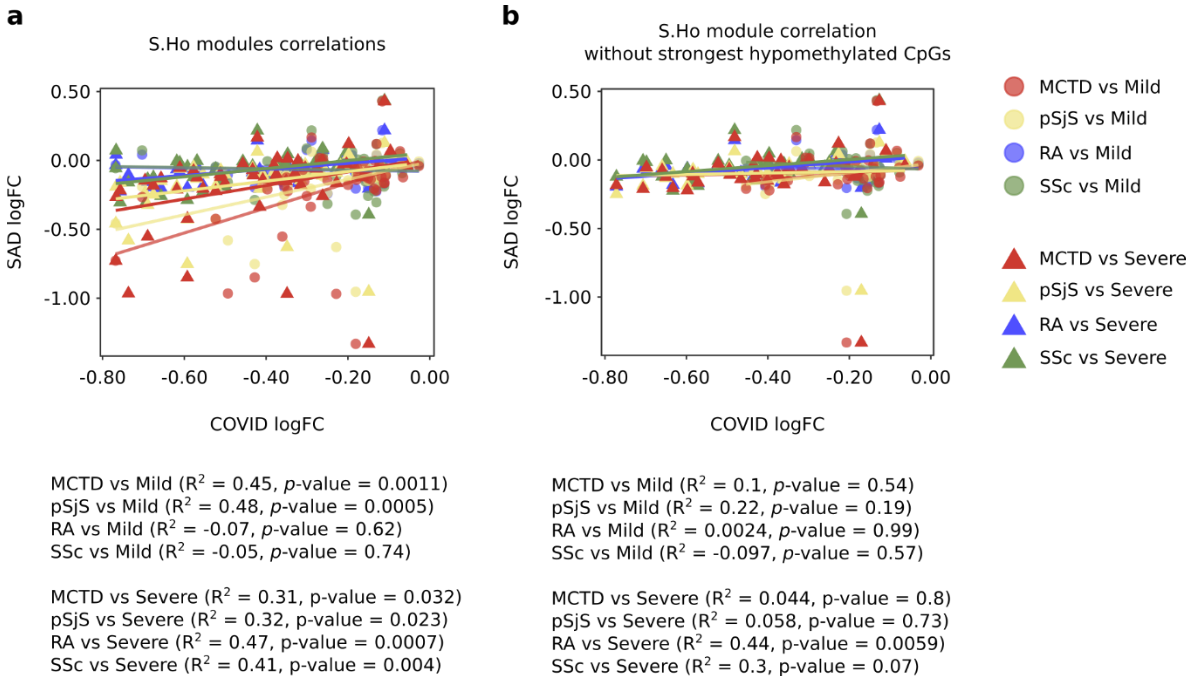

Supplementary Figure 8: Progressive hypomethylation during COVID-19 severity CpG module (S.Ho) is composed of two different functional signatures. (A) logFC correlation plots for severe and mild COVID-19 cases against two interferon related diseases (MCTD and pSjS) and two non-interferon related diseases (RA and SSc) are shown. Correlation coefficients and *p*-values are shown by pairs. (B) logFC correlation plots without strongest hypomethylated CpGs are shown. Circles and triangles represent comparisons against mild and severe cases respectively, and each disease analysis is colored by red (MCTD), yellow (pSjS), blue (RA) and green (SSc).

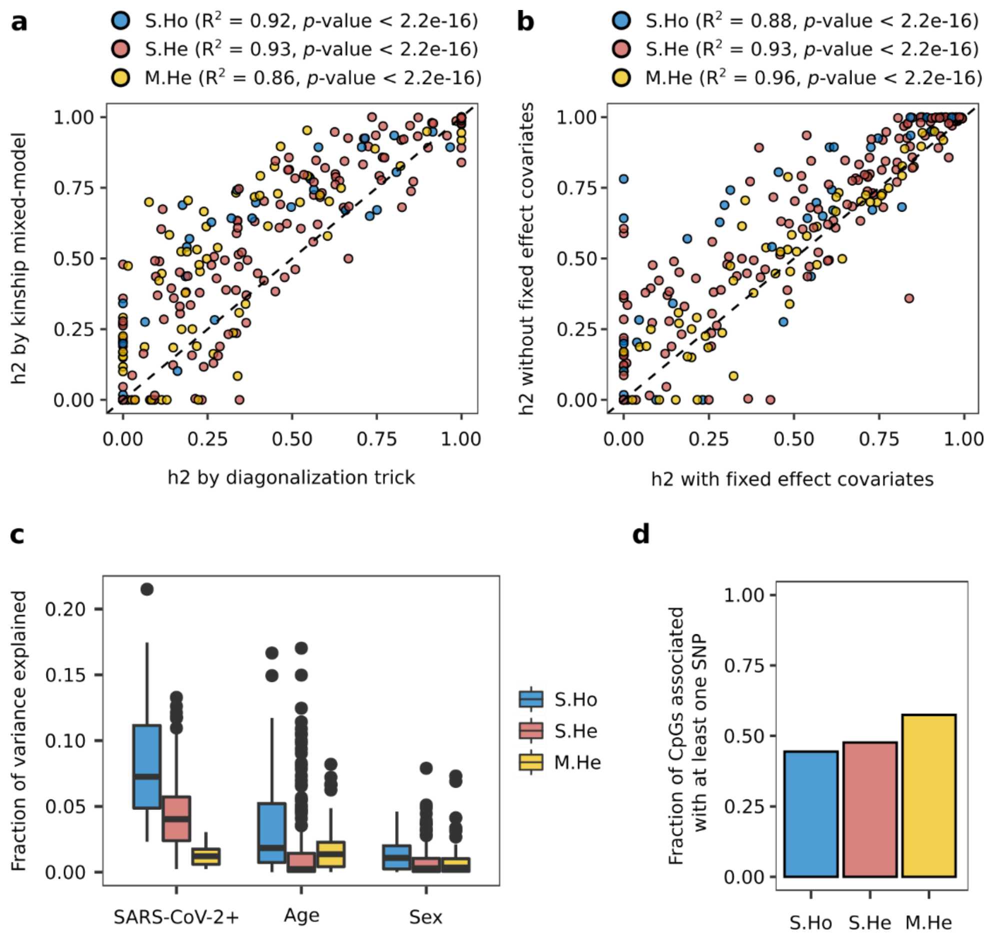

Supplementary Figure 9: Genetic and non-genetic DNA methylation explained variance analyses. (A) Genetic heritability correlation between two independent methods is shown by CpG module (Linear mixed-model variance decomposition and Linear mixed-model fitting with the diagonalization trick were used). Correlation coefficients and *p*-values are depicted by module. (B) Genetic heritability correlation between linear mixed-model variance decomposition with and without fixed effect covariates (SARS-CoV-2 infection, sex and age) is plotted. Correlation coefficients and *p*-values are depicted by module. (C) Fraction of DNA methylation variance explained by fixed effect covariates is shown for each CpG module in all the individuals included in the study (n=574). The center line denotes the median value, the box contains 25th to 75th percentiles of the dataset and the whiskers extend up to ±1.5*IQR. (D) Fraction of CpGs by module associated with at least one SNP is shown. S.Ho module is colored in blue, S.He in red and M.He in yellow.

Supplementary Tables

	WHO scale	Patient state	Descriptor
Negative	0	Uninfected	No clinical or virological evidence of infection
Mild	1	Ambulatory	No limitation of activities
	2		Limitation of activities
	3	Hospitalized mild disease	No oxygen therapy
	4		Oxygen by mask or nasal prongs
Severe	5	Hospitalized severe disease	Non-invasive ventilation or high-flow oxygen
	6		Intubation and mechanical ventilation
	7		Ventilation and additional organ support
	8	Dead	Death

Supplementary Table 1: World Health Organization (WHO) clinical ordinal scale for COVID-19.
