# Supplementary material for: Whole blood DNA methylation analysis reveals respiratory environmental traits involved in COVID-19 severity following SARS-CoV-2 infection
Source: Nat Commun. 2022 Aug 6;13:4597. doi: 10.1038/s41467-022-32357-2 (PMC9357033; doi:10.1038/s41467-022-32357-2)
Supplement: Supplementary file 3 — Description of Additional Supplementary Files [file 41467_2022_32357_MOESM3_ESM.pdf]

Description of Additional Supplementary Files

File Name: Supplementary Data 1
Description: The file includes all statistics from linear regression model performed in the manuscript, including meqtl analysis.

File Name: Supplementary Data 2
Description: Individual collected clinical information.
