# Supplementary material for: Whole blood DNA methylation analysis reveals respiratory environmental traits involved in COVID-19 severity following SARS-CoV-2 infection
Source: Nat Commun. 2022 Aug 6;13:4597. doi: 10.1038/s41467-022-32357-2 (PMC9357033; doi:10.1038/s41467-022-32357-2)
Supplement: Supplementary file 6 — Reporting Summary [file 41467_2022_32357_MOESM6_ESM.pdf]

nature portfolio

Corresponding author(s): Guillermo Barturen & Marta E. Alarcón-Riquelme

Last updated by author(s): Jul 19, 2022

Reporting Summary

Nature Portfolio wishes to improve the reproducibility of the work that we publish. This form provides structure for consistency and transparency in reporting. For further information on Nature Portfolio policies, see our Editorial Policies and the Editorial Policy Checklist.

Statistics

For all statistical analyses, confirm that the following items are present in the figure legend, table legend, main text, or Methods section.

n/a	Confirmed	
☐	☒	The exact sample size (*n*) for each experimental group/condition, given as a discrete number and unit of measurement
☐	☒	A statement on whether measurements were taken from distinct samples or whether the same sample was measured repeatedly
☐	☒	The statistical test(s) used AND whether they are one- or two-sided<br>*Only common tests should be described solely by name; describe more complex techniques in the Methods section.*
☐	☒	A description of all covariates tested
☐	☒	A description of any assumptions or corrections, such as tests of normality and adjustment for multiple comparisons
☐	☒	A full description of the statistical parameters including central tendency (e.g. means) or other basic estimates (e.g. regression coefficient) AND variation (e.g. standard deviation) or associated estimates of uncertainty (e.g. confidence intervals)
☐	☒	For null hypothesis testing, the test statistic (e.g. *F*, *t*, *r*) with confidence intervals, effect sizes, degrees of freedom and *P* value noted<br>*Give P values as exact values whenever suitable.*
☒	☐	For Bayesian analysis, information on the choice of priors and Markov chain Monte Carlo settings
☒	☐	For hierarchical and complex designs, identification of the appropriate level for tests and full reporting of outcomes
☐	☒	Estimates of effect sizes (e.g. Cohen's *d*, Pearson's *r*), indicating how they were calculated

Our web collection on statistics for biologists contains articles on many of the points above.

Software and code

Policy information about availability of computer code

Data collection: No software was used for data collection

Data analysis:
R version 4.1.3
minfi R package (1.42.0)
meffil R package (1.1.2)
missMethyl R package (1.30.0)
EpiDISH R package (2.12.0)
metafor R package (3.4.0)
EPIC array annotation: ilm10b4.hg19 R package (0.6.0)
ReactomePA R package (1.40.0)
HOMER software (4.11)
ssgsea algorithm implemented in escape R package (1.6)
popkin R package (1.3.17)
gaston R package (1.5.7)
MatrixEQTL R package (2.3)
PLINK (1.9 & 2.0)
pheatmap R package (1.0.12)
ggplot2 R package (3.3.6)
ggsci R package (2.9)

For manuscripts utilizing custom algorithms or software that are central to the research but not yet described in published literature, software must be made available to editors and reviewers. We strongly encourage code deposition in a community repository (e.g. GitHub). See the Nature Portfolio guidelines for submitting code & software for further information.

Data

Policy information about availability of data

All manuscripts must include a data availability statement. This statement should provide the following information, where applicable:

- Accession codes, unique identifiers, or web links for publicly available datasets
- A description of any restrictions on data availability
- For clinical datasets or third party data, please ensure that the statement adheres to our policy

Genotypes summary statistics can be accessed through COVID-19 Host Genetic Initiative web page (https://www.covid19hg.org/), included in the project "Determining the Molecular Pathways and Genetic Predisposition of the Acute Inflammatory Process Caused by SARS-CoV-2 (SPGRX)". The genotype data generated (SPGRX cohort) in this study have been deposited in the European Genome-phenome Archive (EGA) database under accession code EGAS00001005304 [https://ega-archive.org/studies/EGAS00001005304]. The methylation data generated in this study have been deposited in the Gene Expression Omnibus (GEO) database under accession code GSE179325 [https://www.ncbi.nlm.nih.gov/geo/query/acc.cgi?acc=GSE179325]. The clinical data collected in this study are provided in the Source Data file (Supplementary Data 2). The additional methylation data used in this study are available in the GEO database under accession code GSE167202 [https://www.ncbi.nlm.nih.gov/geo/query/acc.cgi?acc=GSE167202]. The scRNA-Seq data used in this study are available in the EGA database under accession code EGAS00001004571 [https://ega-archive.org/studies/EGAS00001004571] and in GEO database under accession code GSE158055 [https://www.ncbi.nlm.nih.gov/geo/query/acc.cgi?acc=GSE158055]. EWAS atlas database (https://ngdc.cncb.ac.cn/ewas/atlas), GWAS catalog database (https://www.ebi.ac.uk/gwas/) and COVID-19 Host Genetics Initiative database (https://www.covid19hg.org/) were downloaded on June 2021.

Human research participants

Policy information about studies involving human research participants and Sex and Gender in Research.

Reporting on sex and gender	Sex was obtained from clinical records and the signed informed consent allows sharing this personal information. In the discovery cohort 209 females and 205 males were recruited and in the replication cohort 78 females and 82 males. Sex was included as covariate in the linear regression models.
Population characteristics	All individuals were genetically europeans.<br>Discovery cohort: Negative COVID-19 PCR tested individual had a mean age 63±21 and 43% males, mild COVID-19 patients had a mean age 67±15 and 47% males, severe COVID-19 patients had a mean age 76±14 and 47% males.<br>Replication cohort: Negative COVID-19 PCR tested individual had a mean age 67±20 and 50% males, mild COVID-19 patients had a mean age 61±18 and 53% males, severe COVID-19 patients had a mean age 64±18 and 47% males.
Recruitment	Whole blood was sampled upon arrival to the emergency ward, within a week after first symptoms. Discovery and replication cohorts were recruited between March-April 2020 and August-October 2020, respectively, and from two different clinical centers. No intentional selection was performed on recruited and profiled patients, except for DNA quality, thus we do not expect recruitment/selection related biases that might produce a large impact in the results.
Ethics oversight	The regional ethical committees from Andalucía (Comité Coordinador de Ética de la Investigación Biomédica de Andalucía) and from Valladolid (COMITÉ DE ÉTICA DE LA INVESTIGACIÓN CON MEDICAMENTOS ÁREA DE SALUD VALLADOLID) approved the protocols and gave their ethical approval for this study and all recruited individuals signed the informed consent prior to recruitment.

Note that full information on the approval of the study protocol must also be provided in the manuscript.

Field-specific reporting

Please select the one below that is the best fit for your research. If you are not sure, read the appropriate sections before making your selection.

☒ Life sciences ☐ Behavioural & social sciences ☐ Ecological, evolutionary & environmental sciences

For a reference copy of the document with all sections, see nature.com/documents/nr-reporting-summary-flat.pdf

Life sciences study design

All studies must disclose on these points even when the disclosure is negative.

Sample size	Sample size was not pre-determined, all available individuals with enough DNA quality were included in the analysis.
Data exclusions	Genetic variants and CpG probes not passing the quality controls were discarded.
Replication	The study was performed in two independent cohort of patients, and the results were additionally replicated in an external cohort from a previous publication.
Randomization	Individuals were randomized in technical batches based on severity groups, sex and age.

Blinding	Blinding was not performed nor relevant to this study. We performed a COVID-19 EWAS, where the phenotype needs to be known in advance in order to conduct the analyses.

Reporting for specific materials, systems and methods

We require information from authors about some types of materials, experimental systems and methods used in many studies. Here, indicate whether each material, system or method listed is relevant to your study. If you are not sure if a list item applies to your research, read the appropriate section before selecting a response.

Materials & experimental systems

n/a	Involved in the study
☒	☐ Antibodies
☒	☐ Eukaryotic cell lines
☒	☐ Palaeontology and archaeology
☒	☐ Animals and other organisms
☒	☐ Clinical data
☒	☐ Dual use research of concern

Methods

n/a	Involved in the study
☒	☐ ChIP-seq
☒	☐ Flow cytometry
☒	☐ MRI-based neuroimaging
